# Prognostic Value of the American Heart Association PREVENT Cardiovascular Disease Risk Equations in Cancer Survivorship: A NHANES Population‐Based Study (2009–2018)

**DOI:** 10.1161/JAHA.125.042209

**Published:** 2025-08-10

**Authors:** Mustafa Al‐Jarshawi, Ofer Kobo, Dennis T. Ko, Harindra C. Wijeysundera, M. Golam Azam, Victoria Silverwood, Ram Bajpai, Rodrigo Bagur, Mamas A. Mamas

**Affiliations:** ^1^ Keele Cardiovascular Research Group, Centre for Prognosis Research Keele University Stoke‐on‐Trent United Kingdom; ^2^ Schulich Heart Centre, Sunnybrook Health Sciences Centre University of Toronto Canada; ^3^ The Institute for Clinical Evaluative Sciences (ICES) Toronto Canada; ^4^ Temerty Faculty of Medicine University of Toronto Canada; ^5^ Institute of Health Policy Management and Evaluation University of Toronto Canada; ^6^ National Institute of Cardiovascular Diseases (NICVD) Dhaka Bangladesh; ^7^ School of Medicine Keele University Stoke‐on‐Trent United Kingdom; ^8^ Department of Cardiology, London Health Sciences Centre Western University London Ontario Canada

**Keywords:** baseline cardiovascular risk, cancer survivors, cardiovascular mortality, PREVENT equations all‐cause mortality, Cardiovascular Disease

## Abstract

**Background:**

The PREVENT (Predicting Risk of CVD Events) equations offer a contemporary tool for estimating long‐term cardiovascular risk in the general population. This study evaluates the association of baseline cardiovascular risk, calculated by PREVENT equations, with all‐cause and cardiovascular mortality in cancer survivors.

**Methods:**

Using 10 years of data from the National Health and Nutrition Examination Survey (NHANES) (2009–2018), we analyzed a nationally representative cohort of US cancer survivors. Associations with outcomes were evaluated using Kaplan–Meier curves and multivariable Cox models.

**Results:**

A total of 18 722 334 weighted records (2792 unweighted) were analyzed, recording 4 875 627 all‐cause deaths (26%) and 1 025 053 cardiovascular deaths (5.5%) over a median follow‐up of 9.8 years; 27.84% of cancer survivors were at high baseline cardiovascular risk with variability in baseline cardiovascular risk across different cancer sites. Colon and prostate cancer survivors had the highest prevalence of high cardiovascular risk (54% and 46%, respectively). When compared with low‐risk individuals, those at high cardiovascular risk had a nearly 16‐fold higher risk of all‐cause mortality (adjusted hazard ratio, 15.60 [95% CI, 8.45–28.82]; *P* <0.001) and a 13‐fold higher risk of cardiovascular mortality (adjusted hazard ratio, 12.71 [95% CI, 3.00–53.73]; *P* <0.001) up to a decade of follow‐up. Each 5% increase in baseline cardiovascular risk was associated with higher risks of all‐cause mortality (36%) and cardiovascular mortality (51%) (adjusted hazard ratio, 1.36 [95% CI, 1.30–1.42]; adjusted hazard ratio, 1.51 [95% CI, 1.33–1.72], *P* <0.001 for both).

**Conclusions:**

This study highlights the usefulness of the PREVENT equations for predicting all‐cause and cardiovascular mortality in cancer survivors.

**Nonstandard Abbreviations and Acronyms**
aHR: adjusted hazard ratio
CKM: cardiovascular‐kidney‐metabolic
NHANES: National Health and Nutrition Examination Survey
PREVENT: Predicting Risk of CVD Events

**Clinical Perspective**

**What Is New?**

- This is the first population‐based study to evaluate the prognostic utility of the new American Heart Association's Predicting Risk of CVD Events (PREVENT) equations in cancer survivors using the US National Health and Nutrition Examination Survey.
- A high PREVENT score (≥20%) was associated with up to a 15.6‐fold increase in all‐cause mortality and a 12.7‐fold increase in cardiovascular mortality among US cancer survivors, over up to 10 years of follow‐up.

**What Are the Clinical Implications?**

- The new American Heart Association's PREVENT equations may serve as a prognostic tool for stratifying long‐term mortality risk among cancer survivors.

Advances in the detection and treatment of cancer over the past decade have resulted in improved cancer survival rates and a growing population of cancer survivors. It is estimated that there will be >26 million cancer survivors in the United States by 2040.^1^, ^2^ It is well established that cancer survivors are at increased risk of cardiovascular disease (CVD).^3^, ^4^ with cardiovascular mortality overtaking cancer mortality in some cancer types.^2^ Analogous to the general population, risk assessment for CVD remains fundamental in clinical prevention strategies in this patient population.^5^ Furthermore, professional society guidelines and expert position statements emphasize the need for baseline cardiovascular risk assessment in patients with cancer.^4^, ^6^, ^7^

Traditional CVD risk prediction tools such as the Framingham Risk Score and Pooled Cohort Equations have been widely used in the general population to estimate the 10‐year risk of atherosclerotic cardiovascular disease,^8^ but may not fully capture the unique cardiovascular risk profile of cancer survivors.^9^ The American Heart Association recently introduced the Predicting Risk of CVD Events (PREVENT) equations,^10^ which offer sex‐specific but race‐free 10‐ and 30‐year risk estimates for atherosclerotic cardiovascular disease and heart failure. These equations account for changes in risk factor prevalence across diverse populations, treatment and intervention strategies, and the rising burden of CVD subtypes.^5^

While the PREVENT equations have demonstrated a strong prognostic capability in the general US population,^11^ their prognostic value in cancer survivors remains unexplored. There is a gap in the literature regarding the impact of novel cardiovascular risk assessment tools on overall survival and cardiovascular mortality in this population..^4^ Therefore, this study aims to assess the association between baseline cardiovascular risk, as calculated by this novel tool, and long‐term all‐causes and cardiovascular mortality in cancer survivors. Our findings have the potential to inform tailored cardio‐oncology care and improve long‐term outcomes for cancer survivors at risk of CVD.

## METHODS

All data underlying this article are available in National Health and Nutrition Examination Survey (NAHNES) website, at https://www.cdc.gov/nchs/nhanes/Default.aspx. The data that support the findings of this study are available from the corresponding author upon reasonable request.

### Data Sources

This analysis used data from the National Health and Nutrition Examination Survey (NHANES), which we restricted to adult participants with a diagnosis of cancer. The National Centre for Health Statistics, part of the Centers for Disease Control and Prevention, produces this survey aimed at monitoring the health of the US population. NHANES is a major program that generates a nationally representative sample of the civilian noninstitutionalized US population from ≈5000 individuals each year in a 2‐year cycle. These data are generated through a complex, multistage, probability sampling process with oversampling of specific subgroups to improve the reliability and accuracy of estimates in these populations.^12^ Detailed sampling and data collection procedures have been previously published..^13^ In addition to demographic, socioeconomic, and health‐related questions, the surveys also include medical, physiological, and laboratory measurements.

### Study Design and Population

This study was a retrospective cohort study that used data from the 2009 to 2018 NHANES cycles. Data were linked with the NHANES Linked Mortality File, which links participants from NHANES aged 18 and above with death records in the National Death Index data set through December 31, 2019, the latest mortality data available.^14^

### Study Sample

Survey responders who answered “yes” to the question, “Have you ever been told by a doctor or other health professional that you had cancer or a malignancy of any kind?” in the medical conditions' questionnaire were identified as patients with cancer and included in this analysis. Those identified were further asked, “What kind of cancer?”, and responses were recorded to specify the malignancy organ/site. The cancer organs/site considered in our analysis included hematological (lymphoma and leukemia), bone, brain, breast, genitourinary (kidney, bladder, and prostate), gynecological (ovarian and uterine), gastrointestinal (esophageal, stomach, and colon), liver, gallbladder, pancreas, skin (melanoma, nonmelanoma, and others), soft tissue, and head and neck (thyroid, larynx, mouth, tongue, and lip) cancers. Cancer sites with response rates of <3% were combined into the “other malignancies” category for this analysis.

The total sample of NHANES from 2009 to 2018 comprised 5959 adult participants with questionnaire and laboratory results. Participants were excluded if they were younger than 30 years old, did not have a cancer diagnosis, had a self‐reported history of coronary heart disease, angina, heart attack, or stroke, were ineligible for mortality follow‐up, or had incomplete data for the exposure variable or covariates. The study flowchart is provided in Figure S1.

### Baseline Characteristics

Baseline demographic, socioeconomic, lifestyle, clinical, and laboratory characteristics were collected through standardized questionnaires and objective measurements in Mobile Examination Center assessments. Demographic characteristics included age, sex, and race/ethnicity. Age was recorded as a continuous variable. Sex was classified as male or female, while race and ethnicity were self‐reported and categorized as Mexican American, Other Hispanic, Non‐Hispanic White, Non‐Hispanic Black, and Other Race (including multiracial individuals). Socioeconomic factors included educational attainment and income‐to‐poverty levels. Education level was categorized as Less than High School, High School or Equivalent, or More than High School. The ratio of family income‐to‐poverty level was calculated by dividing family (or individual) income by the poverty guideline specific to the survey year. The midpoint of the reported income range was used to compute the ratio. The poverty income ratio was then stratified into 4 categories: <1.31, 1.31–1.85, 1.86–3.5, and >3.5, based on the US poverty guidelines and federal assistance program eligibility criteria.^15^, ^16^

Smoking status, diabetes, hypertension, hyperlipidemia, and use of anti‐hypertensive and lipid‐lowering medications were also assessed in questionnaires. Diabetes was defined based on a “yes” response to any of the following: “Has a doctor ever told you that you have diabetes?”, “Are you now taking insulin?” or “Are you taking diabetic pills to lower your blood sugar?”. Hypertension was identified through a “yes” response to either “Has a doctor ever told you that you have hypertension?” or “Are you currently taking prescribed medication for hypertension?”. Hyperlipidemia was defined as a “yes” response to either “Has a doctor ever told you that you have a high cholesterol level?” or “Are you now taking prescribed medicine for cholesterol?”. Active smoking was determined based on “Every day” or “Some days” responses to the question, “Do you now smoke cigarettes?”

Systolic blood pressure, height, and weight were measured at the Mobile Examination Center. Body mass index was calculated as weight in kilograms divided by height in meters squared (kg/m^2^). Blood pressure was measured in accordance with NHANES standardized protocols, with 3 consecutive readings recorded and the mean value used for analysis.

Laboratory parameters were obtained from venous blood samples, processed according to NHANES laboratory protocols. The lipid profile was derived from fasting study participants who were examined in the morning session and included total cholesterol (mg/dL) and direct high‐density lipoprotein cholesterol (mg/dL). Glycohemoglobin (%) was measured as an indicator of blood glucose control. Renal function was assessed using serum creatinine (μmol/L) and the estimated glomerular filtration rate (mL/min per 1.73 m^2^) was calculated using the Modification of Diet in Renal Disease formula.^17^

### Exposure

Baseline cardiovascular risk was the primary exposure variable in this study. Baseline cardiovascular risk was calculated using the American Heart Association's PREVENT base equations.^5^ These equations consider the following factors: sex, age, total and high‐density lipoprotein cholesterol levels, estimated glomerular filtration rate, systolic blood pressure, body mass index, diabetes, and smoking status, as well as the use of anti‐hypertensive and lipid‐lowering medications. The PREVENT score categorizes cardiovascular risk into 4 levels: Low Risk (<5%), Borderline Risk (5% to 7.4%), Intermediate Risk (7.5% to 19.9%), and High Risk (≥20%), corresponding to an estimated 10‐year risk of CVD.

### Outcomes

The primary outcome of interest in this study was all‐cause mortality, with a secondary outcome of cardiovascular mortality (*International Statistical Classification of Diseases*, *Tenth Revision* (*ICD*) codes I00–I09, I11, I13, I20‐I51, and I60–I69). Mortality status was assessed via a probabilistic record match to death certificate records from the National Death Index. Additional sources were used to determine mortality status, including those obtained via linkages with the US Social Security Administration and/or by active follow‐up of survey participants. Follow‐up time for each outcome was counted in months from the baseline examination date until the registered date of death or the end of the study (December 31, 2019), whichever occurred first.

### Ethical Approval

The NHANES data set research protocol was approved by the National Center for Health Statistics Ethics Review Board, and all participants signed informed consent. Since the data are publicly available, individual consent for this analysis was not required, and this research was exempt from institutional review board approval. This study adhered to the ethical principles for medical research as outlined in the Declaration of Helsinki.

### Statistical Analysis

All statistical analyses in the main analysis were based on weighted records and conducted using R (R Foundation for Statistical Computing, Vienna, Austria) and SPSS version 28.0.0 (IBM, Armonk, NY). NHANES' weights were applied to account for oversampling, nonresponse, and noncoverage, ensuring nationally representative estimates. Combined weights for the survey cycles 2009 to 2018 were calculated and applied following the NHANES‐recommended formulae for combining weights across the survey cycles. The data‐weighting variable was subsequently rounded to the nearest integer. A detailed explanation of the weighting methods is available on the NHANES website (https://wwwn.cdc.gov/nchs/nhanes/tutorials/weighting.aspx). The normality of data distribution was assessed using the Shapiro–Wilk test. Continuous variables are reported as medians with interquartile ranges, while categorical variables are expressed as proportions. Participants were classified based on baseline cardiovascular risk categories, defined by PREVENT equations. Comparisons between variables were conducted using the design‐based Kruskal–Wallis test for continuous variables and Pearson's χ^2^ test with the Rao & Scott adjustment for categorical variables to account for the complex survey design.

For outcome analysis, we combined the Borderline and Intermediate risk categories into a single Borderline–Intermediate risk group (5%–19.9%) due to low cardiovascular event counts in the Borderline group (Table 1), allowing for sufficiently powered analysis with adequate events per variable and improved clinical interpretability. Survival distributions were assessed using the log‐rank (Mantel–Cox) test and visualized with Kaplan–Meier survival curves. Cox proportional hazards models were used in the main analysis to evaluate the relationship between estimated cardiovascular risk and both all‐cause and cardiovascular mortality. Models were adjusted for the poverty income ratio category, education level, and cancer site. Age, sex, and body mass index were not adjusted for, because these variables were already included in the cardiovascular risk score calculation. Follow‐up time, measured from the NHANES examination date until death or study end (December 31, 2019), was used as the time‐to‐event variable. The proportional hazards assumption for the Cox model was tested using Schoenfeld residuals, confirming no significant violations. Results are reported as adjusted hazard ratios (aHRs) with 95% CIs. All statistical analyses were 2‐tailed, with a *P* value <0.05 considered statistically significant.

**Table 1:** Survey‐Weighted Baseline Characteristics of Study Participants by Baseline Cardiovascular Risk According to PREVENT Equations

Characteristic	Overall N=18 722 334	Low N=5 834 043 (31.16%)*	Borderline N=1 862 598 (9.94%)* ^,^ †	Intermediate N=5 812 854 (31%)*	High N=5 212 840 (27.84%)†	*P* value†
All‐cause mortality	4 875 627 (26%)	304 564 (5.2%)	388 561 (21%)	1 194 340 (21%)	2 988 163 (57%)	<0.001
Cardiovascular mortality	1 025 053 (5.5%)	45 456 (0.8%)	0 (0%)	58 904 (1.0%)	920 694 (18%)	<0.001
Noncardiovascular mortality	3 850 574 (20.5%)	259 108 (4.4%)	388 561 (21%)	1 135 436 (20%)	2 067 469 (39%)	<0.001
Age (y)	64 (53, 75)	48 (36, 54)	58 (54, 60)	67 (63, 72)	80 (76, 80)	<0.001
Sex						<0.001
Men	8 086 481 (43%)	1 071 249 (18%)	1 205 270 (65%)	3 077 311 (53%)	2 732 650 (52%)	
Women	10 635 853 (57%)	4 762 793 (82%)	657 328 (35%)	2 735 543 (47%)	2 480 189 (48%)	
Race/Ethnicity						0.068
Mexican American	436 470 (2.3%)	224 546 (3.8%)	24 825 (1.3%)	108 005 (1.9%)	79 094 (1.5%)	
Other Hispanic	338 855 (1.8%)	211 976 (3.6%)	55 813 (3.0%)	35 925 (0.6%)	35 140 (0.7%)	
Non‐Hispanic White	16 622 281 (89%)	4 791 720 (82%)	1 661 596 (89%)	5 360 890 (92%)	4 808 074 (92%)	
Non‐Hispanic Black	896 477 (4.8%)	323 306 (5.5%)	120 364 (6.5%)	162 276 (2.8%)	290 531 (5.6%)	
Other	428 251 (2.3%)	282 494 (4.8%)	0 (0%)	145 757 (2.5%)	0 (0%)	
Ratio of family income to poverty						<0.001
<1.31	2 487 894 (13%)	856 764 (15%)	68 459 (3.7%)	713 277 (12%)	849 393 (16%)	
1.31–1.85	1 560 477 (8.3%)	413 903 (7.1%)	21 743 (1.2%)	563 064 (9.7%)	561 766 (11%)	
1.86–3.5	4 361 467 (23%)	765 010 (13%)	349 358 (19%)	1 258 024 (22%)	1 989 075 (38%)	
>3.5	10 312 496 (55%)	3 798 365 (65%)	1 423 037 (76%)	3 278 488 (56%)	1 812 605 (35%)	
Education level						0.040
Less than High School	3 086 543 (16%)	817 698 (14%)	148 397 (8.0%)	1 002 886 (17%)	1 117 563 (21%)	
High school or equivalent	3 776 805 (20%)	766 258 (13%)	317 884 (17%)	1 280 589 (22%)	1 412 073 (27%)	
More than High school	11 858 985 (63%)	4 250 087 (73%)	1 396 317 (75%)	3 529 379 (61%)	2 683 203 (51%)	
Systolic blood pressure (mm Hg)	124 (112, 136)	114 (106, 124)	120 (110, 134)	126 (114, 138)	136 (124, 150)	<0.001
Body mass index (kg/m^2^)	28 (24, 33)	27 (24, 33)	28 (25, 33)	29 (24, 34)	28 (24, 31)	0.6
Total cholesterol (mg/dL)	191 (165, 219)	192 (166, 221)	191 (158, 228)	202 (177, 227)	180 (155, 208)	0.001
Direct HDL cholesterol (mg/dL)	51 (42, 65)	56 (45, 66)	48 (40, 60)	50 (42, 68)	51 (40, 62)	0.2
GFR (mL/min)	89 (66, 109)	106 (91, 140)	98 (81, 123)	84 (68, 105)	60 (45, 74)	<0.001
Glycohemoglobin (%)	5.60 (5.30, 6.00)	5.30 (5.20, 5.60)	5.50 (5.30, 5.70)	5.80 (5.50, 6.10)	5.80 (5.50, 6.30)	<0.001
Active smoking	2 415 228 (13%)	1 137 310 (19%)	371 732 (20%)	624 323 (11%)	281 863 (5.4%)	0.006
Hypertension medication	7 834 640 (42%)	829 405 (14%)	461 261 (25%)	2 669 833 (46%)	3 874 141 (74%)	<0.001
Hyperlipidemia medication	5 381 873 (29%)	429 655 (7.4%)	522 267 (28%)	2 186 820 (38%)	2 243 131 (43%)	<0.001
Hyperlipidemia	8 218 876 (50%)	1 419 250 (31%)	818 576 (45%)	3 190 193 (61%)	2 790 857 (58%)	0.001
Hypertension	9 199 546 (49%)	1 446 937 (25%)	720 444 (39%)	2 947 894 (51%)	4 084 272 (78%)	<0.001
Diabetes	2 979 472 (16%)	163 139 (2.8%)	27 119 (1.5%)	1 178 643 (20%)	1 610 571 (31%)	<0.001
Cancer site						0.025
Breast	2 330 094 (12%)	469 564 (8.0%)	203 473 (11%)	926 618 (16%)	730 438 (14%)	
Cervix	1 658 291 (8.9%)	1 152 755 (20%)	78 077 (4.2%)	296 026 (5.1%)	131 432 (2.5%)	
Colon	1 154 471 (6.2%)	191 595 (3.3%)	91 373 (4.9%)	250 311 (4.3%)	621 193 (12%)	
Melanoma	891 882 (4.8%)	344 435 (5.9%)	57 399 (3.1%)	207 468 (3.6%)	282 580 (5.4%)	
Prostate	1 695 376 (9.1%)	298 369 (5.1%)	70 991 (3.8%)	548 467 (9.4%)	777 549 (15%)	
Skin (others)	6 619 445 (35%)	1 816 354 (31%)	760 567 (41%)	2 515 424 (43%)	1 527 100 (29%)	
Uterus	597 856 (3.2%)	239 214 (4.1%)	91 014 (4.9%)	212 110 (3.6%)	55 519 (1.1%)	
Others	3 774 919 (20%)	1 321 756 (23%)	509 704 (27%)	856 429 (15%)	1 087 029 (21%)	

Unweighted total sample size=2792. Unweighted baseline characteristics by baseline cardiovascular risk according to PREVENT (Predicting Risk of CVD Events) equations are presented in Table S1. GFR indicates glomerular filtration rate.

* Median (Q1, Q3); n (%). N (%).

^†^ Design‐based Kruskal–Wallis test; Pearson's χ^2^: Rao & Scott adjustment.

Unweighted baseline characteristics and estimates are presented in the Supplementary Material. Additionally, we explored the association between estimated cardiovascular risk and both cardiovascular and noncardiovascular mortality using Fine and Gray competing risk models, ^18^, ^19^, applied to both the weighted and unweighted samples. Results were reported as subdistribution hazard ratios as part of a supplementary analysis.

## RESULTS

A total of 2792 unweighted cancer survivors (aged 30 to 80 years) were included in the analysis, representing 18 722 334 individuals after applying survey weights. All participants had complete vital status data.

### Baseline Characteristics

The risk distribution showed 5 834 043 (31.16%) participants at low cardiovascular risk, 1 862 598 (9.94%) at borderline risk, 5 812 854 (31%) at intermediate risk, and 5 212 840 (27.84%) at high cardiovascular risk. Individuals in the high cardiovascular risk category were older, with a median age of 80 years, compared with those at intermediate (67 years), borderline (58 years), and low cardiovascular risk (48 years). Participants at low cardiovascular risk were more likely to be female (82% versus 18% male) and more likely to be Mexican American or Other Hispanic (3.8% and 3.6%, respectively) compared with non‐Hispanic Whites (82%) and non‐Hispanic Blacks (5.5%). They also were more frequently active smokers (19%) than their higher‐risk counterparts (5.4%). Conversely, these participants were less likely to have diabetes, hypertension, or hyperlipidemia (Table 1). The observed differences between those groups showed significant differences across several characteristics, as outlined in Table 1. Corresponding unweighted descriptive statistics are provided in Table S1.

Table 2 presents the distribution of baseline cardiovascular risk according to the PREVENT equations by cancer site in NHANES participants. Of note, 70% of participants with cervical cancer demonstrated low CVD risk, the highest percentage within this category, while colon cancer had the highest proportion of participants with high CVD risk at 54%, followed by prostate cancer at 46%. Uterine cancer participants had the highest proportion of borderline cardiovascular risk at 15%, while breast cancer had the highest proportion of intermediate cardiovascular risk at 40%. The unweighted distributions are presented in Table S2.

**Table 2:** Survey‐Weighted Distribution of Baseline Cardiovascular Risk According to PREVENT Equations by Cancer Site

Risk category†	Overall N=18 722 334*	Breast N=2 330 094*	Cervix N=1 658 291*	Colon N=1 154 471*	Melanoma N=891 882*	Prostate N=1 695 376*	Skin (others) N=6 619 445*	Uterus N=597 856*	Others N=3774919*
Low	5 834 043 (31%)	469 564 (20%)	1 152 755 (70%)	191 595 (17%)	344 435 (39%)	298 369 (18%)	1 816 354 (27%)	239 214 (40%)	1 321 756 (35%)
Borderline	1 862 598 (9.9%)	203 473 (8.7%)	78 077 (4.7%)	91 373 (7.9%)	57 399 (6.4%)	70 991 (4.2%)	760 567 (11%)	91 014 (15%)	509 704 (14%)
Intermediate	5 812 854 (31%)	926 618 (40%)	296 026 (18%)	250 311 (22%)	207 468 (23%)	548 467 (32%)	2 515 424 (38%)	212 110 (35%)	856 429 (23%)
High	5 212 840 (28%)	730 438 (31%)	131 432 (7.9%)	621 193 (54%)	282 580 (32%)	777 549 (46%)	1 527 100 (23%)	55 519 (9.3%)	1 087 029 (29%)

* N (%).

^†^ Pearson's *X* ^2^: Rao & Scott adjustment; Design‐based Kruskal–Wallis test (*P*‐value=0.025).

### RESULTS

Over a median follow‐up of 118 months, 4 875 627 all‐cause deaths (26%) and 1 025 053 cardiovascular deaths (5.5%) were recorded in the weighted data set. Over up to 10 years of follow‐up, we observed that mortality rates were highest among individuals at high cardiovascular risk (57% all‐cause, 18% cardiovascular mortality), compared with intermediate cardiovascular risk (21% all‐cause, 1.0% cardiovascular mortality), borderline cardiovascular risk (21% all‐cause, 0% cardiovascular mortality), and low cardiovascular risk (5.2% all‐cause, 0.8% cardiovascular mortality). Corresponding unweighted mortality rates across cardiovascular risk categories are reported in Table S1.

The Figure presents the weighted Kaplan–Meier survival curves for all‐cause and cardiovascular mortality among cancer survivors, stratified by estimated baseline cardiovascular risk categories according to the PREVENT equations. Survivors with high baseline cardiovascular risk had lower survival probabilities than those with borderline–intermediate or low baseline cardiovascular risk (all‐cause mortality: log‐rank=109.1, *P*<0.001; cardiovascular mortality: Log‐Rank=59.4, *P* <0.001). Survival disparities widened over time, with the greatest differences emerging at longer follow‐up durations. Survival curves by estimated baseline cardiovascular risk categories for each cancer site are presented in Figure S2.

**Figure 1:**
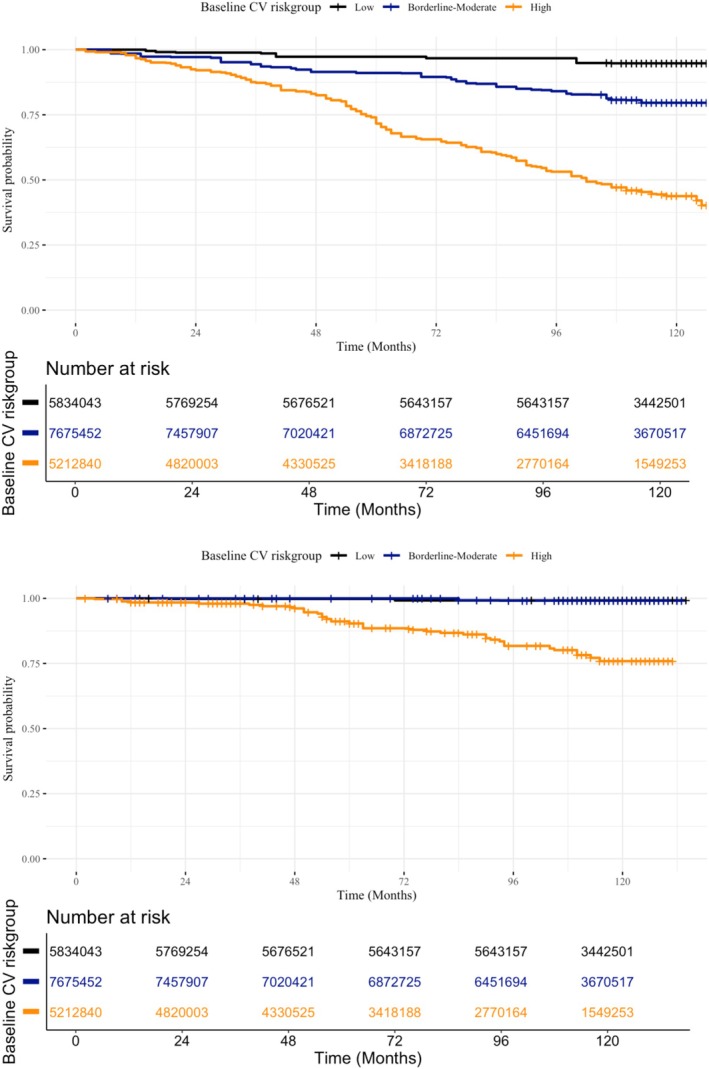
Survey‐weighted Kaplan–Meier survival curves by baseline cardiovascular risk categories according to PREVENT equations in National Health and Nutrition Examination Survey (2013–2018) cancer population for all‐cause mortality, and cardiovascular mortality. *P* value for log‐rank (<0.01). CV indicates cardiovascular.

Table 3 presents the association between baseline cardiovascular risk categories according to the PREVENT equations and survival outcomes, using the Cox proportional hazards regression model for both all‐cause and cardiovascular mortality. Given the differences in social determinants of health characteristics presented in Table 1, both models were adjusted for poverty income ratio and education levels. Compared with individuals at low baseline cardiovascular risk, those in the borderline–intermediate risk category had an aHR of 4.38 for all‐cause mortality (95% CI, 2.48–7.74, *P*<0.001) but did not show a significant difference in cardiovascular mortality risk (aHR, 0.65 [95% CI] 0.09–4.64, *P*=0.66). Individuals at high cardiovascular risk had an aHR of 15.60 for all‐cause mortality (95% CI, 8.45–28.82, *P*<0.001) and an aHR of 12.71 for cardiovascular mortality (95% CI, 3.00–53.73, *P*<0.001). Corresponding unweighted hazard ratio estimates are presented in Table S3.

**Table 3:** Survey‐Weighted Multivariable‐Adjusted Hazard for All‐Cause and Cardiovascular Mortality Associated With Baseline Cardiovascular Risk According to PREVENT Equations

All‐Cause Mortality		
Baseline cardiovascular risk category	Events	aHR
Low	304 564 (5.33)	Reference
Borderline–intermediate	1 582 901 (21.07)	4.38
High	2 988 163 (58.39)	15.60

aHR indicates adjusted hazard ratio, adjusted to the ratio of family income to poverty category, education level, and cancer site. Events, number of events; in parentheses, event rate per 1000 person‐years. PREVENT indicates Predicting Risk of CVD Events.

In a continuous model, each 5% increase in the baseline cardiovascular risk according to the PREVENT equations was associated with a 36% higher risk of all‐cause mortality (aHR, 1.36 [95% CI, 1.30–1.42]; *P* <0.001) and a 51% increased risk of cardiovascular mortality (aHR, 1.51 [95% CI, 1.33–1.72]; *P* <0.001), as shown in (Table 4). Unweighted estimates for this analysis are provided in Table S4.

**Table 4:** Survey‐Weighted Multivariable‐Adjusted HR for All‐Cause & Cardiovascular Mortality Associated With Every 5% Increase in Baseline PREVENT Score

Variable	All‐cause mortality			Cardiovascular mortality		
	aHR	95% CI	*P* value	aHR	95% CI	*P* value
Per 5% increase in PREVENT Score	1.36	1.30–1.42	<0.001	1.51	1.33–1.72	<0.001

Adjusted to the ratio of family income to poverty category, education level, and cancer site. aHR indicates adjusted hazard ratio; and CI, confidence interval.

### Supplementary Analysis

Table S5 presents the association between baseline cardiovascular risk and noncardiovascular mortality using Fine and Gray competing risk models. Compared with individuals at low cardiovascular risk, those in the borderline–intermediate and high‐risk categories had a significantly increased risk of noncardiovascular mortality, with subdistribution hazards ratio of 4.82 (95% CI, 2.41–9.63, *P* <0.001) and 11.58 (95% CI, 5.29–25.32, *P* <0.001), respectively. Corresponding unweighted estimates are reported in Table S5.

Table S6 shows, consistent with the main analysis, a persistent association between high baseline cardiovascular risk and increased cardiovascular mortality in the competing‐risk models. Compared with individuals at low cardiovascular risk, those in the high‐risk category had a markedly increased risk of cardiovascular mortality (subdistribution hazards ratio, 14.01 [95% CI, 3.37–58.26]; *P* <0.001). Additionally, each 5% increase in the PREVENT score was significantly associated with higher cardiovascular mortality (subdistribution hazards ratio, 1.50 [95% CI, 1.35–1.66]; *P* <0.001). Corresponding unweighted estimates are reported in Table S6.

## DISCUSSION

The present study aimed to assess the association between baseline cardiovascular risk according to the PREVENT equations and long‐term all‐cause and cardiovascular mortality among cancer survivors over a 10‐year follow‐up period. Our analysis of a large cohort of patients with cancer in NHANES cycles (2009–2018), representing >18 million US cancer survivors, demonstrated several key findings with significant implications for cardiovascular risk stratification and mortality prediction in this population. We found that a quarter of cancer survivors had a high baseline cardiovascular risk. Cancer survivors at high cardiovascular risk were older and had more comorbidities than those at lower risk, consistent with existing literature on the impact of age and comorbidities on cardiovascular risk in cancer survivors.^20^ We observed notable variation in baseline cardiovascular risk across different cancer sites. Colon and prostate cancer survivors had the highest prevalence of high cardiovascular risk, respectively, whereas cervical cancer survivors, who tend to be younger, had the highest prevalence of low cardiovascular risk. These findings highlight substantial variability in cardiovascular risk distribution among different cancer sites, likely influenced not only by age at diagnosis but also by shared risk factors such as hypertension, obesity, and chronic inflammation, emphasizing the need for tailored cardiovascular risk assessment in cancer survivors.

Survival analysis demonstrated an incremental increase in mortality risk associated with higher baseline cardiovascular risk defined by the PREVENT equations. While the PREVENT tool is primarily designed to predict CVD and its components, our findings suggest it may also have relevance in understanding broader mortality risks in cancer survivors. When compared with cancer survivors at low cardiovascular risk, those at high cardiovascular risk as estimated by the PREVENT equations had a 15.6‐fold higher risk of all‐cause mortality and a 12.7‐fold higher risk of cardiovascular mortality during up to a decade of follow‐up. Every 5% increase in estimated 10‐year cardiovascular risk was associated with a 36% increase in the risk of all‐cause mortality and 51% increase in the risk of cardiovascular mortality. These findings align with the recent external validation study of the PREVENT equations by Scheuermann et al, which demonstrated that a 1% increase in PREVENT risk estimates was associated with a 9% higher risk of cardiovascular mortality (HR, 1.09 [95% CI, 1.087–1.094]) in the general population.^11^ The shorter follow‐up time for participants from more recent NHANES cycles (eg, 2017–2018) may have influenced the magnitude of observed survival differences and may have partially contributed to the observed widening of survival disparities over time.

The higher cardiovascular mortality rates (18%) in cancer survivors at high baseline cardiovascular risk, compared with those at intermediate (1.0%), borderline (0%), and low cardiovascular risk (0.8%), further highlight the need for tailored and proactive cardiovascular risk management in this group to ensure that reductions in cancer mortality, likely achieved through advances in cancer diagnosis and treatments, are not offset by an increase in cardiovascular mortality over the long term. Interestingly, active smoking was more prevalent among individuals at low and borderline cardiovascular risk compared with those at high cardiovascular risk. This finding may represent an opportunity to optimize cardiovascular risk management through targeted smoking cessation efforts and early preventive interventions. It may also reflect survivorship bias, where cancer survivors in higher cardiovascular risk categories had a lower prevalence of active smoking, possibly due to increased mortality among smokers.

Unlike traditional CVD risk prediction tools, the new PREVENT equations incorporate impaired cardiovascular‐kidney‐metabolic (CKM) health into CVD risk assessment. CKM health reflects the pathophysiological interactions between metabolic risk factors (such as obesity, diabetes, and chronic kidney disease) and the cardiovascular system.^21^ These conditions frequently co‐occur and disproportionately affect underrepresented groups in the population..^22^, ^23^, ^24^, ^25^ Traditional risk tools such as the Framingham Risk Score and Pooled Cohort Equations significantly underestimate atherosclerotic cardiovascular disease risk in cancer survivors, particularly those who undergo treatments such as radiation therapy.^9^ This is likely because these models fail to account for CKM health factors and do not adjust for competing risks of non‐CVD deaths, which have major implications for morbidity and mortality in cancer survivors..^21^, ^26^ In contrast, the PREVENT equations integrate CKM factors and adjust for competing risks, enhancing their applicability in this population.

Our findings have significant implications for clinical practice and public health, particularly in improving survival and reducing disparities among cancer survivors. The utility of the PREVENT equations to predict all‐cause and cardiovascular mortality risks specifically in cancer survivors had not been previously explored. This study highlights the prognostic utility of the PREVENT equations in identifying cancer survivors at increased risk for adverse mortality outcomes.

### Study Strengths

The strengths of this population‐based study include the use of a large, nationally representative sample of cancer survivors in the United States, which facilitates the external validity of our findings to US cancer survivors and the long‐term follow‐up period of up to 10 years. Furthermore, we were able to control for cancer site and potential demographic confounding factors not considered within the PREVENT equations such as the ratio of family income to poverty and education levels. The analysis stratified cardiovascular risk across different cancer sites, highlighting the variability in cardiovascular risk distribution and emphasizing the need for tailored risk assessments.

### Study Limitations

First, the observational design of our study excludes causal inference, and residual confounding may have influenced the results despite adjustments for relevant covariates. Second, the reliance on self‐reported data in NHANES questionnaires for certain variables may introduce bias and affect the accuracy of the findings.^27^ Third, the exclusion of participants with incomplete data could bias the results and limit generalizability. Although using the PREVENT basic equations limited the number of excluded records, these equations did not incorporate additional CKM factors such as hemoglobin A1c, urine albumin‐to‐creatinine ratio, and social deprivation index, which are currently under development.^10^ Fourth, the relatively small sample size within certain cancer sites may have limited the statistical power of some of our findings. Fifth, we lack information on guideline‐directed medical treatment for primary or secondary cardiovascular risk prevention, cancer‐specific medications, and cardiovascular‐ or cancer‐specific interventions, all of which would have impacted on the outcomes of interest. Sixth, reliance on NHANES mortality data, which may sometimes be subject to miscoding, could affect the validity of the mortality outcomes. Finally, the NHANES‐linked NDI database includes only mortality events, which limited our ability to evaluate the association between the PREVENT tool and long‐term nonfatal cardiovascular events in cancer survivors. Future research with larger, more diverse cohorts, including characterization by cancer histology or stage, specific cancer therapies, and longitudinal follow‐up, is needed to further validate our findings and explore potential mechanisms underlying the observed differences in cardiovascular risk and mortality outcomes in our study.

## CONCLUSIONS

This study supports the use of the PREVENT equations in stratifying cardiovascular risk across different cancer sites and predicting long‐term all‐cause and cardiovascular mortality among cancer survivors at high cardiovascular risk. While baseline estimated cardiovascular risk varies across different cancer sites, the association between baseline cardiovascular risk and mortality appears consistent across these groups. These findings provide strong evidence supporting the use of the PREVENT score as a reliable predictor of mortality outcomes in cardio‐oncology, thus offering equitable cardiovascular risk assessment and management strategies tailored to diverse cancer survivor populations.

## Sources of Funding

None.

## Disclosure

V. Silverwood is currently funded by the School of Primary Care Research (SPCR).

## Supporting information

Tables S1–S6
Figures S1–S2
