# Supplementary material for: Prognostic Value of the American Heart Association PREVENT Cardiovascular Disease Risk Equations in Cancer Survivorship: A NHANES Population‐Based Study (2009–2018)
Source: J Am Heart Assoc. 2025 Aug 10;14(20):e042209. doi: 10.1161/JAHA.125.042209 (PMC12684629; doi:10.1161/JAHA.125.042209)

Supplemental Materials

Table S1: unweighted baseline characteristics of study participants by baseline CV risk according to PREVENT equations						
**Characteristic**	**Overall**<br>N = 2,792†	**Low**<br>N = 653†	**Borderline**<br>N = 212†	**Intermediate**<br>N = 838†	**High**<br>N = 1,089†	**p-value**
**All-cause mortality**	898 (32%)	42 (6.4%)	56 (26%)	183 (22%)	617 (57%)	<0.001
**CV mortality**	252 (9.0%)	12 (1.8%)	0 (0%)	19 (2.3%)	221 (20%)	<0.001
**Non-CV mortality**	646 (23%)	30 (4.6%)	56 (26%)	164 (20%)	396 (36%)	
**Age (years)**	69 (56, 79)	44 (31, 52)	58 (54, 60)	68 (63, 73)	80 (75, 80)	<0.001
**Gender**						<0.001
Men	1,346 (48%)	137 (21%)	128 (60%)	430 (51%)	651 (60%)	
Women	1,446 (52%)	516 (79%)	84 (40%)	408 (49%)	438 (40%)	
**Race/Ethnicity**						0.068
Mexican American	182 (6.5%)	72 (11%)	16 (7.5%)	50 (6.0%)	44 (4.0%)	
Other Hispanic	130 (4.7%)	43 (6.6%)	22 (10%)	35 (4.2%)	30 (2.8%)	
Non-Hispanic White	2,140 (77%)	454 (70%)	140 (66%)	639 (76%)	907 (83%)	
Non-Hispanic Black	280 (10%)	53 (8.1%)	34 (16%)	85 (10%)	108 (9.9%)	
Other Race	60 (2.1%)	31 (4.7%)	0 (0%)	29 (3.5%)	0 (0%)	
**Ratio of family income to poverty**						<0.001
<1.31	624 (22%)	192 (29%)	9 (4.2%)	182 (22%)	241 (22%)	
1.31-1.85	281 (10%)	66 (10%)	13 (6.1%)	94 (11%)	108 (9.9%)	
1.86-3.5	742 (27%)	101 (15%)	65 (31%)	186 (22%)	390 (36%)	
>3.5	1,145 (41%)	294 (45%)	125 (59%)	376 (45%)	350 (32%)	
**Education level**						0.040
Less than High School	656 (23%)	133 (20%)	44 (21%)	177 (21%)	302 (28%)	
High school or equivalent	657 (24%)	124 (19%)	33 (16%)	227 (27%)	273 (25%)	
More than High school	1,479 (53%)	396 (61%)	135 (64%)	434 (52%)	514 (47%)	
**Systolic blood pressure (mm Hg)**	126 (114, 138)	116 (106, 124)	124 (114, 138)	126 (116, 138)	136 (124, 150)	<0.001
**Body Mass Index (kg/m**2)**	28 (24, 32)	27 (24, 33)	28 (25, 33)	29 (25, 34)	28 (24, 31)	<0.001
**Total Cholesterol (mg/dL)**	188 (162, 214)	194 (173, 218)	196 (158, 221)	200 (175, 226)	178 (152, 205)	<0.001
**Direct HDL-Cholesterol (mg/dL)**	51 (42, 62)	55 (43, 64)	49 (42, 67)	51 (42, 65)	51 (41, 61)	<0.001
**GFR (ml/min)**	81 (61, 105)	109 (95, 142)	98 (81, 118)	86 (69, 105)	58 (44, 73)	<0.001
**Glycohemoglobin (%)**	5.70 (5.40, 6.10)	5.40 (5.20, 5.70)	5.60 (5.30, 5.90)	5.80 (5.50, 6.10)	5.80 (5.50, 6.40)	<0.001
**Active smoking**	380 (14%)	163 (25%)	46 (22%)	103 (12%)	68 (6.2%)	0.006
**Hypertension medication**	1,332 (48%)	94 (14%)	63 (30%)	398 (47%)	777 (71%)	<0.001
**Hyperlipidemia medication**	895 (32%)	43 (6.6%)	74 (35%)	337 (40%)	441 (40%)	<0.001
**Hyperlipidemia**	1,285 (53%)	171 (35%)	96 (47%)	471 (63%)	547 (55%)	<0.001
**Hypertension**	1,568 (56%)	208 (32%)	81 (38%)	452 (54%)	827 (76%)	<0.001
**Diabetes Mellitus**	548 (20%)	28 (4.3%)	3 (1.4%)	173 (21%)	344 (32%)	<0.001
**Cancer site**						<0.001
Breast	381 (14%)	77 (12%)	22 (10%)	150 (18%)	132 (12%)	
Cervix	210 (7.5%)	135 (21%)	12 (5.7%)	38 (4.5%)	25 (2.3%)	
Colon	218 (7.8%)	25 (3.8%)	7 (3.3%)	60 (7.2%)	126 (12%)	
Melanoma	128 (4.6%)	26 (4.0%)	10 (4.7%)	27 (3.2%)	65 (6.0%)	
Prostate	390 (14%)	35 (5.4%)	15 (7.1%)	113 (13%)	227 (21%)	
Skin (others)	739 (26%)	139 (21%)	40 (19%)	263 (31%)	297 (27%)	
Uterus	127 (4.5%)	46 (7.0%)	44 (21%)	29 (3.5%)	8 (0.7%)	
Others	599 (21%)	170 (26%)	62 (29%)	158 (19%)	209 (19%	

†Median (Q1, Q3); n (%).

Table S2: unweighted distribution of baseline CV risk according to PREVENT equations by cancer site									
**Risk category**	**Overall**<br>N = 2,792†	**Breast**<br>N = 381†	**Cervix**<br>N = 210†	**Colon**<br>N = 218†	**Melanoma**<br>N = 128†	**Prostate**<br>N = 390†	**Skin (others)**<br>N = 739†	**Uterus**<br>N = 127†	**Others**<br>N = 599†
Low	653 (23%)	77 (20%)	135 (64%)	25 (11%)	26 (20%)	35 (9.0%)	139 (19%)	46 (36%)	170 (28%)
Borderline	212 (7.6%)	22 (5.8%)	12 (5.7%)	7 (3.2%)	10 (7.8%)	15 (3.8%)	40 (5.4%)	44 (35%)	62 (10%)
Intermediate	838 (30%)	150 (39%)	38 (18%)	60 (28%)	27 (21%)	113 (29%)	263 (36%)	29 (23%)	158 (26%)
High	1,089 (39%)	132 (35%)	25 (12%)	126 (58%)	65 (51%)	227 (58%)	297 (40%)	8 (6.3%)	209 (35%)

†n (%).

Table S3: unweighted multivariable-adjusted HR for all-cause & CV mortality associated with baseline CV risk according to PREVENT equations						
	**All-Cause Mortality**			**Cardiovascular Mortality**		
**Baseline CV risk category**	**aHR**[1]	**95% CI**†	**p-value**	**aHR**†	**95% CI**†	**p-value**
Low	Reference	-	-	Reference	-	-
Borderline-intermediate	4.46	2.55- 7.79	<0.001	0.66	0.09- 4.76	0.68
High	14.76	8.39- 25.97	<0.001	13.51	3.20- 57.09	<0.001

†aHR = Adjusted Hazard Ratio, CI = Confidence Interval. Adjusted to – the ratio of family income to poverty category, education level, and cancer site.

Table S4: unweighted multivariable-adjusted HR for all-cause & CV mortality associated with every 5% increase in baseline PREVENT score

	All-Cause Mortality			Cardiovascular Mortality		
**Variable**	**aHR**[†]	**95% CI**[†]	**p-value**	**aHR**[†]	**95% CI**[†]	**p-value**
**Per 5% increase in PREVENT Score**	1.41	1.31- 1.51	<0.001	1.52	1.33– 1.74	<0.001

[†]aHR = Adjusted Hazard Ratio, CI = Confidence Interval. Adjusted to –the ratio of family income to poverty category, education level, and cancer site.

Table S5: Multivariable sub-distribution HR for non-CV mortality associated with baseline CV risk according to PREVENT equations

	Unweighted			Weighted		
**Baseline CV risk category**	**sHR**[†]	**95% CI**[†]	**p-value**	**sHR**[†]	**95% CI**[†]	**p-value**
Low	Reference	-	-	Reference	-	-
Borderline-intermediate	4.69	1.97- 11.17	<0.001	4.82	2.41- 9.63	<0.001
High	9.06	3.90- 21.04	<0.001	11.58	5.29- 25.32	<0.001

[†] sHR= subdistribution Hazard Ratio, CI = Confidence Interval. Adjusted to – the ratio of family income to poverty category, education level, and cancer site.

Table S6: Multivariable sub-distribution HR for CV mortality associated with baseline CV risk according to PREVENT equations

	Unweighted			Weighted		
**Baseline CV risk category**	**sHR**[†]	**95% CI**[†]	**p-value**	**sHR**[†]	**95% CI**[†]	**p-value**
Low	Reference	-	-	Reference	-	-
Borderline-intermediate	0.90	0.14- 5.50	0.91	0.93	0.16- 5.56	0.94
High	13.50	3.19- 57.00	<0.001	14.01	3.37- 58.26	<0.001
**Per 5% increase in PREVENT Score**	1.48	1.33- 1.65	<0.001	1.50	1.35- 1.66	<0.001

[†] sHR= subdistribution Hazard Ratio, CI = Confidence Interval. Adjusted to – the ratio of family income to poverty category, education level, and cancer site.

Figure S1: Study flowchart

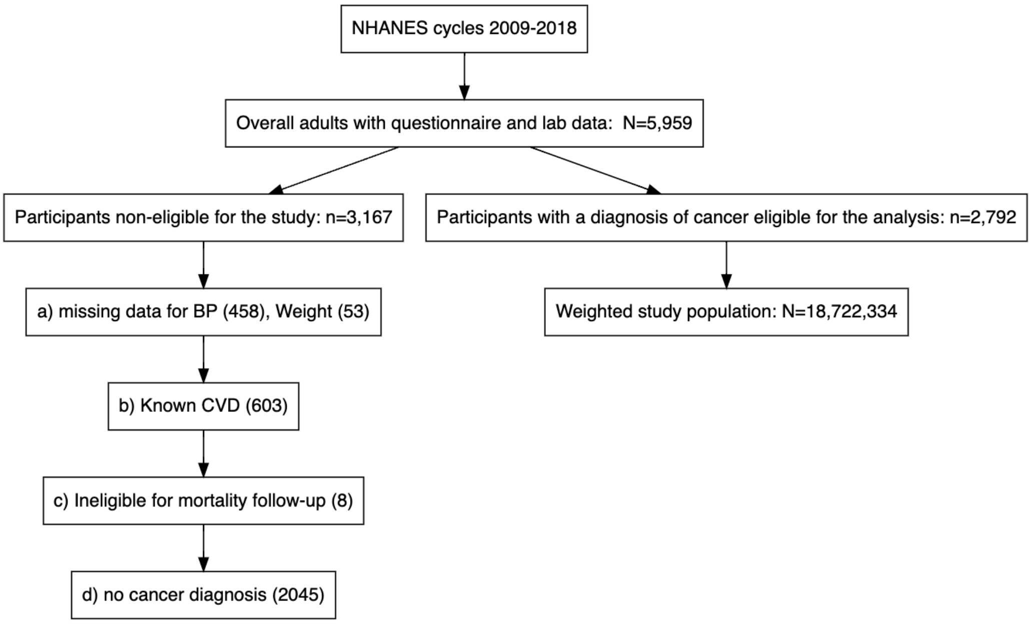

Figure S2: Survey-weighted Kaplan–Meier (KM) Survival Curves by Baseline CV risk Categories According to PREVENT Equations, Stratified by Cancer Site, for a) All-cause mortality, and b) CV mortality.

a) All-cause mortality

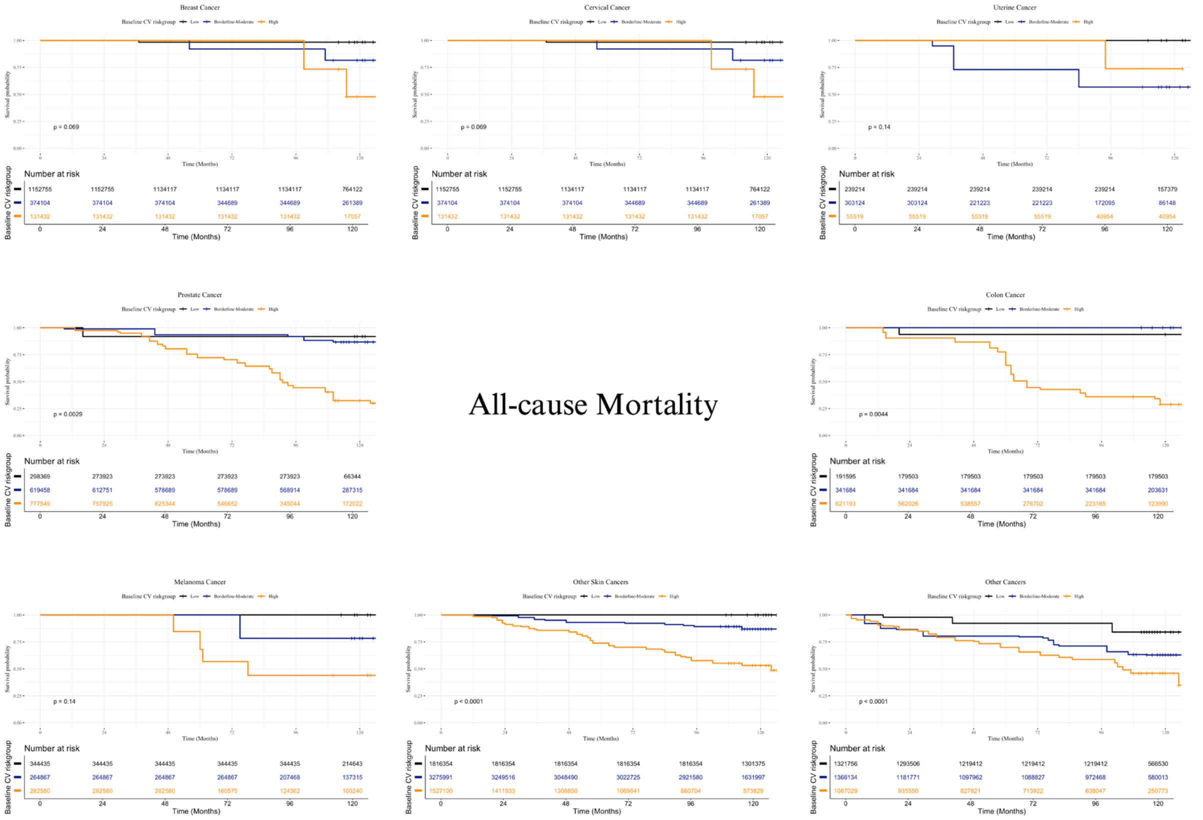

b) CV mortality

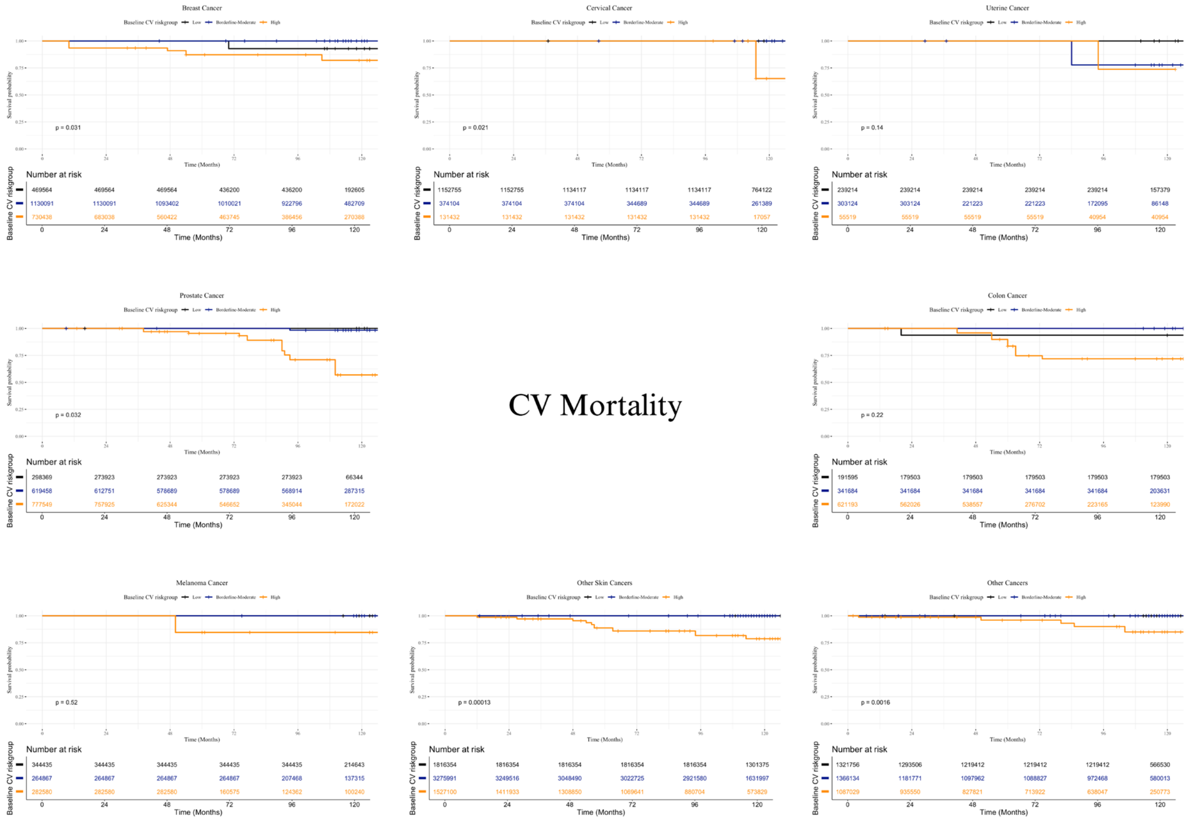

Supplement: Supplementary file 1 — Tables S1–S6 Figures S1–S2 [file JAH3-14-e042209-s001.pdf]
